# A multidisciplinary approach to verify and de-label of drug allergic histories in a university hospital in Thailand: a retrospective descriptive study

**DOI:** 10.1186/s40545-023-00513-8

**Published:** 2023-01-20

**Authors:** Sirinoot Palapinyo, Jettanong Klaewsongkram, Pungjai Mongkolpathumrat, Nattawut Leelakanok, Kitiyot Yotsombut

**Affiliations:** 1 Department of Pharmacy Practice, Faculty of Pharmaceutical Sciences, Chulalongkorn University, Bangkok, 10330 Thailand; 2 Center of Excellence in Bioactive Resources for Innovative Clinical Applications, Chulalongkorn University, Bangkok, Thailand; 3 Division of Allergy and Clinical Immunology, Department of Medicine, Faculty of Medicine, Chulalongkorn University, Bangkok, Thailand; 4 King Chulalongkorn Memorial Hospital, Thai Red Cross Society, Bangkok, Thailand; 5 The Skin and Allergy Research Unit, Chulalongkorn University, Bangkok, Thailand; 6 Division of Clinical Pharmacy, Faculty of Pharmaceutical Sciences, Burapha University, Chonburi, Thailand

**Keywords:** Drug allergy, ADR, De-labeling, Multidisciplinary

## Abstract

**Background:**

Mislabeling of drug allergic histories causes avoidable negative impacts on patients and healthcare system. Although multidisciplinary adverse drug reaction (ADR) services to verify and de-label drug allergic histories have been operated in particular hospitals in Thailand, their performances have not been reported. This research aimed to examine the effectiveness of verification of drug allergic history and de-labeling (VD) services of the physician-led multidisciplinary ADR clinic.

**Methods:**

This research was a retrospective descriptive study. Medical charts of patients with at least one drug allergic history who received VD services at the multidisciplinary clinic between January 2017 to December 2018, were reviewed. Data on the history of drug allergy, VD services, and results were analyzed and presented using descriptive statistics.

**Results:**

Seventy patients’ charts were reviewed, and 171 unconfirmed drug allergic histories were identified. 79.53% of the reported reactions involved skin and soft tissues. The most found adverse skin reactions were maculopapular rash, pruritic and erythematous rash, and angioedema. The remaining 20.47% were systemic reactions which included drug reaction with eosinophilia and systemic symptoms (DRESS), anaphylaxis, and nausea/vomiting was the most prevalent. Antituberculosis, beta-lactam antibiotics, and nonsteroidal anti-inflammatory drugs (NSAIDs) were the most reported suspected drugs. Drug allergic history reviewing by physicians or pharmacists could confirm and de-label for 3 and 20 reactions, respectively. Seven and one reactions were confirmed by enzyme‐linked immunospot assay and patch test, respectively. The provocation tests with the suspected or alternative drug were conducted in 64 reactions. Twelve reactions were confirmed, and 45 reactions were de-labeled. Totally, 65/171 (38.01%) allergic histories were successfully de-labeled, 23/171 (13.45%) were confirmed, and 83/171 (48.53%) were inconclusive.

**Conclusions:**

More than half of drug allergic histories were successfully confirmed or de-labeled by the multidisciplinary ADR team. The collaborative activities of various healthcare professionals, consisting of physicians, nurse, and pharmacists as presented in the study were effective in VD services and should be implemented in other healthcare settings.

## Background

Adverse drug reaction (ADR) is a collective term used to describe a significantly harmful or unpleasant reaction that potentially results from medications [1]. Drug toxicity, side-effect, drug allergy, idiosyncratic reaction, teratogenesis, carcinogenesis, and drug withdrawal all fall under the umbrella of ADR. ADR that is not related to dose and pharmacological actions of the drug includes immunological, and idiosyncratic reactions [2]. The immunological involvement of the reaction renders the term ‘drug allergy’ or ‘drug hypersensitivity’ to be used. The prevalence of reported penicillin allergy ranges from 5% in the general population to 15% in hospitalized patients[3]. NSAID hypersensitivity has been reported among 0.6–2.5% of the general population and may reach 5–10% in patients with bronchial asthma or other underlying atopic conditions [4]. To deal with drug allergy, withholding and avoiding the use of the culprit drugs are the proper management to prevent further harmful results from re-exposure to the drug [5].

Avoidance of drug allergy by not using the culprit or suspected drugs requires the record and reconciliation of drug allergic history in all relevant clinical databases including hospital databases, private clinic databases, drugstore databases, and other healthcare facilities’ databases. The patient should also be informed and educated to avoid the culprit or suspected drugs as well as to notify healthcare professionals regarding his or her allergic history when receiving any medications. These recordings and informing are known as ‘labeling’ of drug allergic history. Although labeling helps prevent repeated exposure to the allergic drug, labeling can have a negative impact directly on the patient as well as on the healthcare system [6]. A systematic review found that penicillin allergy labeling is related to the use of broader-spectrum or second-line antibiotics, higher cost of treatment, longer hospitalization, higher readmission rates, and a higher rate of antimicrobial resistance [7]. Drug allergy mislabeling leads to higher costs of treatment and unnecessary limited use of drugs which sometimes can be life-threatening [8, 9]. Several studies have identified the prevalence of drug allergy mislabeling and found that the prevalence has ranged between 0.32% and 27.35% [10–12]. The high variability in the prevalence of drug allergy mislabeling encourages the investigation of such prevalence locally to add more understanding to the issue. The drug allergic history reported by Thai patients or their caregivers is at high risk of errors for various reasons: (1) drug allergy is sometimes diagnosed by the patient (self-diagnosis), family members, or non-qualified healthcare professionals, (2) lack of accurate and complete information for making the right diagnosis, and (3) misunderstanding, confusion, or inability to remember the drug name, due to language barrier are commonly found among Thai patients [13].

Verification of drug allergic history and de-labeling (VD) services have been shown to effectively remove the inaccurate labeling of drug allergic history, and thus could aid antibiotic stewardship and rational use of medications [14, 15]. The VD services involve several complex steps: (1) comprehensive reviewing of allergic history to drugs and any chemicals, (2) identifying an immediate allergic reaction by skin prick and intradermal tests, (3) identifying a non-immediate reaction by patch tests, and (4) confirming the allergic reaction by drug provocation tests [16]. Therefore, cooperative roles of a multidisciplinary care team with differing expertise including physicians, nurses, and pharmacists are required [17]. In Thailand, the multidisciplinary VD services are limited to only particular tertiary-level hospitals or university hospitals due to insufficient human and laboratory resources. The data regarding the effectiveness of such team services in Thailand's context has not been examined. This study aimed to investigate the prevalence of drug allergic history mislabeling among Thai patients and examine the effectiveness of VD services of the multidisciplinary ADR professionals in Thailand.

## Methods

### Study design

This retrospective descriptive study was conducted in a physician-led multidisciplinary ADR clinic. Patient information recorded in the hospital databases from January 2017 to December 2018 was reviewed. All ambulatory and in-patients with at least one drug allergic history recorded in the databases who were referred to the clinic for VD services were included in the study. The research protocol was approved by the Institutional Review Board of the Faculty of Medicine, Chulalongkorn University (Approval Number 777/63).

**Fig. 1:**
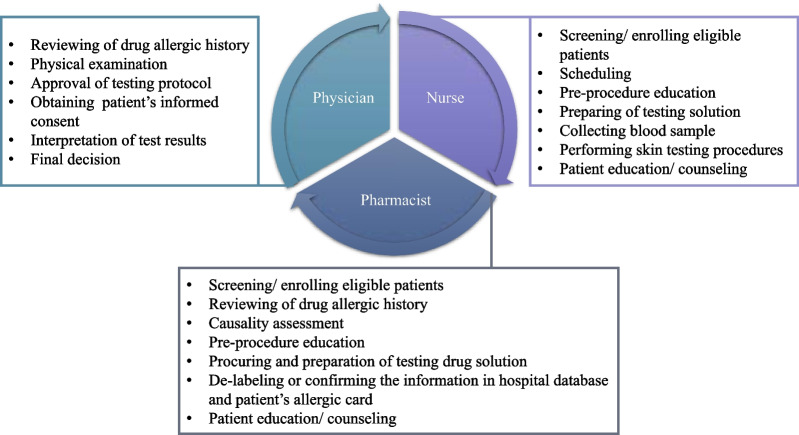
Roles and responsibilities of the multidisciplinary team members

The clinic was specialized for patients with positive histories of drug allergy or other forms of ADRs. The members of the clinic were two immunology physicians, one dermatology physician, one certified nurse specialized in allergy testing, and three clinical pharmacists specialized in ADRs. The clinic has been operated under an internal medicine department of a university hospital in Thailand. The VD services were available every Tuesday and Thursday from 9 a.m. to 4 p.m. The roles and responsibilities of team members regarding VD services are shown in Table 1 and Fig. 1. Working experiences as health care providers and as an ADR care team of the clinic members were 16.5 (interquartile range: 10.0–25.0) years and 5.5 (interquartile range: 2.0–12.0) years, respectively.

**Table 1:** Roles and responsibilities of the multidisciplinary team members

	Activities	Physician	Nurse	Pharmacist
1	Initial screening and enrolling of eligible patients		/	/
2	Reviewing and verification of drug allergic history	/		/
3	In vitro testing	/	/	/
4	Skin testing	/	/	/
5	Drug provocation testing	/	/	/
6	De-labeling or confirmation of drug allergic history	/		/

### Drug allergic history VD services

Patients with unconfirmed drug allergic history were identified and referred to the multidisciplinary ADR clinic by any physicians in the hospital. At the clinic, the patients were screened and enrolled in VD services by the nurse or the pharmacist. The enrolled patients were then interviewed, and their medical records were reviewed by the physician or the pharmacist to verify the patients’ drug allergic history. The Naranjo’s algorithm was used for the causality assessment [18]. In this step, de-labeling occurred when the experienced adverse reactions were compatible with other alternative diagnoses, i.e., drug intolerance or side effects.

The remaining patients received a battery of in vitro tests for drug allergy. In some patients, multiple tests were performed. Drug-specific interferon-gamma (IFN-γ)-releasing cells by enzyme‐linked immunospot assay (IFN-γ ELISpot assay, Mabtech, Stockholm, Sweden), ELISpot, was used in patients with non-immediate reactions or unclear history. A patch test (Finn Chambers, Hyrylia, Finland) was also used in patients with non-immediate reactions. In patients with immediate reactions, a skin prick test, followed by an intradermal (ID) test were considered. The test solutions used in the skin prick test and ID test were freshly prepared from the suspected drugs by the pharmacists according to available published protocols [19]. Blood sample collections and skin testing procedures were performed by the nurse.

Provocation test, the controlled administration of the suspected drug to diagnose drug allergy, was used when the previous diagnostic evaluations were negative or unavailable. The provocation test was not considered in pregnant patients, patients with severe, life-threatening immunocytotoxic reactions, e.g., severe cutaneous adverse reactions (SCARs), vasculitis syndromes, and exfoliative dermatitis, since these conditions were contraindicated [20].

### Data collection and statistical analysis

Data on the history of drug allergy including the name of the suspected drug, characteristics of the reaction, age at onset of the reaction; demographics of the patients including current age, and underlying illness; and data regarding the VD services including the causality assessment, laboratory results, skin test results, and provocation tests were collected. The data were analyzed with Microsoft Excel, using descriptive statistics.

## Results

Seventy patients were referred to the clinic for VD services between January 2017 and December 2018. Data from a total number of 70 patients were reviewed and analyzed. The characteristics of the included patients are presented in Table 2. Most of the included patients were non-pregnant females (64.29%). The median age of the included patients was 51 (interquartile range [IQR]: 36–63) years. Almost all the patients (92.86%) had an underlying illness. Cardiovascular diseases were the common underlying diseases found in patients (Table 2).

**Table 2:** Characteristics of the included patients

Patient characteristics	All patients (*N* = 70)
*Gender*	
Male—no. (%)	25 (35.71)
Female—no. (%)	45 (64.29)
Median age at the encounter (Interquartile range: IQR)—year	51 (36–63)
Median age when the allergy occurred (IQR)—year	45 (25–55)*
Median duration with the allergy history (IQR)—year	1 (0–7)*
Total number of allergic history—item	171
Skin and soft tissue reactions—no. (%)	136 (79.53)
Systemic reactions—no. (%)	35 (20.47)
Median number of drug allergic history (IQR)—items/person	2 (1–3)
History of food allergy—no. (%)	4 (5.7)
Concomitant diseases—no. (%)	65 (92.86)
Cardiovascular diseases	32 (45.71)
Cancer	17 (24.29)
Asthma and/or allergic rhinitis	12 (17.14)
Chronic kidney disease	11 (15.17)
Bone and joint diseases	9 (12.86)
Human Immunodeficiency Virus infection	8 (11.43)
Opportunistic infection including tuberculosis	8 (11.43)
Diabetes mellitus	7 (10.00)
Hepatitis and chronic liver diseases	6 (8.57)
Neurological and psychiatric disorders	6 (8.57)
Skin diseases	6 (8.57)
Systemic lupus erythematosus	4 (5.71)
Others	4 (5.71)

^*^In case that a patient reported more than one allergic reaction, the earliest age was used for the calculation

The median number of drug allergic histories was 2 (IQR: 1–3) reactions/patient. A total of 171 unconfirmed drug allergic reactions received VD services (Tables 2 and 3). One hundred and thirty-six reactions (79.53%) involved skin and soft tissues. Maculopapular rash, pruritic and erythematous rash, and angioedema were the most prevalent skin reactions, which accounted for 23.53%, 16.18%, and 9.56% of all skin reactions, respectively. Among 35 systemic reactions, drug reaction with eosinophilia and systemic symptoms (DRESS), anaphylaxis, and nausea/vomiting were the most found (45.71%, 14.29%, and 14.29%, respectively). Beta-lactam antibiotics, anti-tuberculosis, and nonsteroidal anti-inflammatory drugs (NSAIDs) were the most reported suspected drugs (21.63%, 14.62%, and 9.94%, respectively).

**Table 3:** Drug groups and associated adverse reactions recorded in the patient charts

Reported Adverse reaction*	Antimicrobial agents										Anti-cancer			Cardiovascular drugs				
	Antifungal drugs	Anti-tuberculosis	Beta lactams	Clindamycin	Doxycycline	Fluoroquinolones	Macrolides	Oseltamivir	Sulfa antibiotics	Vancomycin	EGFR–TKIs	Nivolumab	Platinum-based antineoplastic drugs	ACE inhibitors	CCB	Clopidogrel	Diuretics	Statins
*Skin and soft tissue reactions*																		
Acneiform dermatitis	**–**	–	1	–	–	–	–	–	–	–	–	–	–	–	–	–	–	–
AGEP	1	–	2	1	–	–	–	–	–	1	–	–	–	–	–	–	–	–
Angioedema	–	–	3	1	–	–	–	–	–	–	–	–	–	–	–	–	–	–
Bullous pemphigoid	–	–	–	–	–	–	–	–	–	–	–	1	–	–	–	–	–	–
Eczematous eruption	–	3	–	–	–	–	–	–	–	–	–	–	–	–	–	–	–	–
Edema	–	–	–	–	–	–	–	–	–	–	–	–	–	–	1	–	–	–
Erythema multiforme	–	–	4	–	–	1	–	1	1	–	–	–	–	–	–	–	–	–
Exfoliative dermatitis	1	4	1	–	–	–	–	–	2	–	–	–	–	–	–	–	–	–
Fixed drug eruption	–	–	–	–	–	–	–	–	2	–	–	–	–	–	–	–	–	–
MP rash	–	8	8	–	–	2	–	–	2	2	–	–	–	–	1	–	–	–
Mucositis	–	–	1	–	–	–	–	–	–	–	–	–	–	–	–	–	–	–
PRIDE	–	–	–	–	–	–	–	–	–	–	11	–	–	–	–	–	–	–
Pruritic and erythematous rash	–	–	9	2	–	3	4	–	–	–	–	–	–	1	–	–	–	–
SDRIFE	–	–	1	–	–	–	–	–	–	–	–	–	–	–	–	–	–	–
SJS	1	4	1	–	–	–	–	–	1	–	–	–	–	–	–	–	–	–
TEN	1	–	1	–	–	–	–	–	1	–	–	–	–	–	–	–	–	–
Urticaria	–	–	1	2	–	–	1	–	–	–	–	–	–	–	–	–	–	–
*Systemic reactions*																		
Anaphylaxis	–	–	1	–	–	–	–	–	–	–	–	–	2	–	–	–	–	–
DRESS	–	4	1	–	–	–	–	–	3	–	–	–	–	–	–	–	–	–
Dyspnea	–	–	2	–	–	–	–	–	–	–	–	–	–	–	–	–	–	–
Hepatitis	–	2	–	–	–	–	–	–	–	–	–	–	–	–	–	–	–	1
Hypertension	–	–	–	–	–	–	–	–	–	–	–	–	–	–	–	–	–	–
Nausea and vomiting	–	–	–	–	1	2	–	–	–	–	–	–	–	–	–	–	–	–
Syncope	–	–	–	–	–	–	–	–	–	–	–	–	–	–	–	1	–	–
Thrombocytopenia	–	–	–	–	–	–	–	–	–	–	–	–	–	–	–	–	1	–
Total	4	25	37	6	1	8	5	1	12	3	11	1	2	1	2	1	1	1

Reported Adverse reaction*	Drugs for pain control							Miscellaneous						Total
	Uric lowering agents	Antiepileptics	Codeine	Local anesthetics	Muscle relaxant	NSAIDs	Pregabalin	Antihistamines	Contrast media	Flupentixol plus melitracen	Food supplement	Immunosuppressants	Pseudoephedrine	
*Skin and soft tissue reactions*														
Acneiform dermatitis	–	–	–	–	–	–	–	–	–	–	–	–	–	1
AGEP	–	–	–	–	–	–	–	–	–	–	–	1	–	6
Angioedema	–	–	–	–	1	7	–	–	–	–	1	–	–	13
Bullous pemphigoid	–	–	–	–	–	–	–	–	–	–	–	–	–	1
Eczematous eruption	–	–	–	–	–	–	–	–	–	–	–	–	–	3
Edema	–	–	–	–	–	–	1	–	1	–	–	1	–	4
Erythema multiforme	–	–	–	–	–	–	–	–	–	–	1	–	–	8
Exfoliative dermatitis	–	–	–	–	–	–	–	–	–	–	–	–	–	8
Fixed drug eruption	–	–	–	–	–	–	–	2	–	–	–	–	–	4
MP rash	3	1	1	–	–	2	–	1	–	–	–	1	–	32
Mucositis	–	–	–	–	–	–	–	–	–	–	–	–	–	1
PRIDE	–	–	–	–	–	–	–	–	–	–	–	–	–	11
Pruritic and erythematous rash	–	–	–	–	–	–	–	–	–	–	–	1	2	22
SDRIFE	–	–	–	–	–	–	–	–	–	–	–	–	–	1
SJS	–	–	2	–	–	–	–	–	–	–	–	–	–	9
TEN	–	–	–	–	–	–	–	–	–	–	–	–	–	3
Urticaria	–	–	–	–	–	4	–	–	–	1	–	–	–	9
*Systemic reactions*														
Anaphylaxis	–	–	–	–	1	–	–	–	–	–	–	1	–	5
DRESS	2	–	5	–	–	1	–	–	–	–	–	–	–	16
Dyspnea	–	–	–	–	–	–	–	–	–	–	–	–	–	2
Hepatitis	–	–	–	–	–	1	–	–	–	–	–	–	–	4
Hypertension	–	–	–	1	–	–	–	–	–	–	–	–	–	1
Nausea and vomiting	–	–	–	–	–	2	–	–	–	–	–	–	–	5
Syncope	–	–	–	–	–	–	–	–	–	–	–	–	–	1
Thrombocytopenia	–	–	–	–	–	–	–	–	–	–	–	–	–	1
Total	5	1	8	1	2	17	1	3	1	1	2	5	2	171

^*^ AGEP: acute generalized exanthematous pustulosis; CCB: calcium channel blockers; DRESS: drug reaction with eosinophilia and systemic symptoms; MP rash: maculo-papular rash; PRIDE: papulopustules and/or paronychia, regulatory abnormalities of hair growth, itching, and dryness due to epidermal growth factor receptor inhibitors; SDRIFE: symmetrical drug-related intertriginous and flexural exanthema; SJS: Stevens–Johnson syndrome; TEN: toxic epidermal necrolysis

**Fig. 2:**
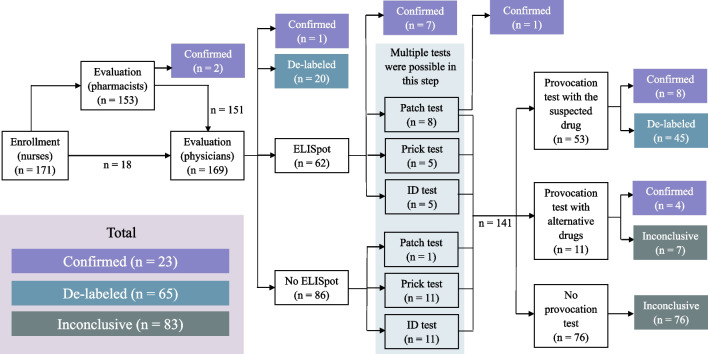
Multidisciplinary verification and de-labeling services and outcomes

Of 171 unconfirmed drug allergic reactions at the enrollment, 153 reactions were evaluated by the pharmacists. The Naranjo’s algorithm was applicable only in 140 allergic reactions, since insufficient data were found in the other 13 reactions. The causal relationships between the suspected drug and its adverse reaction were possible for 87 reactions, probable for 52 reactions, and doubtful for 1 reaction. However, 2 confirmed allergic reactions were considered by global clinical judgment because of strongly suggestive histories. The remaining 169 reactions were then evaluated by the physicians. One allergic reaction was confirmed, and 20 reactions were successfully de-labeled (Fig. 2).

Sixty-two reactions were eligible for ELISpot, yielding 7 confirmed allergic reactions. Further various skin testing procedures were performed. Multiple tests for one reaction were possible in this step. The patch test, skin prick test, and ID test were conducted for 9, 16, and 16 tests, respectively. Only one allergic reaction was confirmed by the patch test. Therefore, 141 reactions were spared for the provocation tests (Fig. 2).

For the provocation tests, 53 reactions were tested with the suspected drugs. Confirmed and de-labeled allergic reactions were 8 and 45 reactions, respectively. Eleven reactions were tested with alternative, chemically similar drugs instead of the suspected drugs. Four reactions, compatible with the reported histories, were observed and confirmed. Although 7 reactions were not observed with the alternative drug, de-labeling was not possible, because the suspected drugs were not tested. Thus, those 7 reactions were classified as inconclusive. The remaining 76 reactions did not receive any provocation tests and were classified as inconclusive as well (Fig. 2).

As a result, the multidisciplinary ADR team was able to de-label 65 drug allergic histories (38.01%) and confirm 23 allergic reactions (13.45%). Counseling regarding allergic history confirmation or de-labeling was delivered to all patients. The hospital databases, patient records, and drug allergy personal cards were successfully corrected. The remaining 83 reactions (48.53%) were inconclusive, so the allergic histories were not altered.

## Discussion

The physician-led multidisciplinary team in this study was able to identify and de-label the mislabeled drug allergic histories for 38.01%, which was higher than the prevalence of mislabeling reported in previous literatures (0.32–27.35%) [11, 12, 16]. However, almost half of allergic reactions were inconclusive. This was not surprising, since the diagnosis of drug allergy can be challenging, especially with limited information regarding the reported allergic reactions. Insufficient reliable history and records, language barrier, confusion about drug allergy and other forms of adverse reactions, attitudes toward drug allergy and safety of the de-labeling processes, and recall biases were commonly observed in this study. The Naranjo’s algorithm was not so helpful in those cases. As a result, only 3 (1.75%) and 20 (11.70%) reactions were confirmed and de-labeled based on reviewing and verification of allergic history by physicians or pharmacists. There was a limited number of reactions receiving skin testing in our study. This was because of (1) unavailable standard or published protocols for the suspected drugs, (2) the suspected drugs were not suitable for skin testing due to their direct irritation or cytotoxic properties, and (3) the allergic reactions were unlikely to obviously respond with skin testing, e.g., DRESS, acneiform eruptions, acute generalized exanthematous pustulosis (AGEP), hepatitis, and thrombocytopenia [21, 22]. About half of the reactions in this study were not eligible for the provocation test, because the patient’s conditions or drug allergic reactions were contraindicated, such as pregnancy, SCARs, or other life-threatening reactions [20]. Although some reactions in particular patients were good candidates, those patients denied the provocation test.

In our study, there were 11 provocation tests that used alternative drugs instead of the suspected drug. This was because of unavailability of the suspected drug or other compelling reasons such as searching for another safer cephalosporin for the ongoing infections in patients with ceftriaxone allergic histories [23, 24]. The four observed reactions, which were similar to the reported reactions, were confirmed as true allergic history. On the contrary, we could not de-label the seven reactions with negative results from provocation tests using the alternative drugs, since they possibly were real ones, but very specific to the culprit drugs. Even though those reactions were labeled as inconclusive with a need for further investigation, usable alternative drugs for such patients were identified.

The mislabeling of drug allergic history was still a challenging problem among Thai patients. Such problems were not limited to only antibiotics but also found in other drug classes. Besides, the successful de-labeling required sophisticated actions and procedures of various expertise rather than based solely on history reviewing or laboratory testing. Therefore, the value of the multidisciplinary ADR team, consisting of different healthcare professionals with cooperative roles and responsibilities, was clearly demonstrated in our study. However, there was a high proportion of inconclusive reactions that could not be exactly determined due to insufficient information or other reasons as mentioned. Some of them may not be the problem if they were systematically investigated, completely documented, and judiciously considered by experienced healthcare professionals at the time of adverse reaction occurrence, since all necessary data could possibly be completed [25]. Measures to improve patients’ knowledge and understanding of drug allergy such as comprehensive counseling with printed information personalized for patients' health literacy levels and a validated drug allergy personal card should also be effectively provided [26].

This study has several limitations that need to be clarified. First, the study subjects were purposefully referred to the ADR clinic by other departments of the hospital. Those patients were initially screened by other healthcare professionals, who tended to report multiple drug allergic reactions (the median number of drug allergic reactions was 2 items/person), and had higher demand for de-labeling or confirmation of drug allergic history, because there was no available treatment for their conditions. The provocation tests with the suspected drugs or the alternative drugs were also performed at a higher rate in our study than the usual care in routine situations. Second, the reported drug allergic reactions in our study comprised different adverse reactions with various suspected drug classes. Subgroup analysis based on drug class or type of ADR was not performed, because insufficient sample size could lead to inaccurate interpretation. Finally, individual reported reactions were not tested with all available tests, especially the provocation test. Therefore, the true prevalence of mislabeling as well as the performance of the aforementioned in vitro or skin tests were not possible to be determined.

Future studies should be conducted in a larger group of general populations with various health statuses and different drug allergic histories, so that the study could be more generalizable and applicable for subgroup analysis. It should also be designed to ensure that all reported reactions will be examined by all possible testing methods, including the provocation test to determine the real prevalence and performance of each test. Moreover, the impact of de-labeling, such as the acceptability of patients and caregivers, further allergic reaction to the de-labeled drug, and clinical, humanistic, and economic outcomes of the services, should be studied.

## Conclusions

Mislabeling of drug allergic history was commonly found among Thai patients. Such histories were successfully de-labeled by the multidisciplinary team. Confirmation of drug allergic history or identification of safe alternative drugs for individual patients by the team were also helpful. Those findings suggest that the collaborative activities of various healthcare professionals as the multidisciplinary ADR team in this study should be widely implemented in healthcare settings.

## Data Availability

All data generated or analyzed during this study are included in this published article.

## Abbreviations

ADR: Adverse drug reaction
DRESS: Drug reaction with eosinophilia and systemic symptoms
ELISpot: Enzyme‐linked immunospot
IFN: Interferon
ID: Intradermal
NSAIDs: Nonsteroidal anti-inflammatory drugs
SCARs: Severe cutaneous adverse reactions
VD: Verification of drug allergic history and de-labeling

## References

[1] Khalil H, Huang C (2020). Adverse drug reactions in primary care: a scoping review. BMC Health Serv Res.

[2] Smith W (2013). Adverse drug reactions—allergy? side-effect? intolerance?. Aust Fam Physician.

[3] Steenvoorden L, Bjoernestad EO, Kvesetmoen T-A, Gulsvik AK (2021). De-labelling penicillin allergy in acutely hospitalized patients: a pilot study. BMC Infect Dis.

[4] Bangerl T, Zahel B, Lueger A, Guenova E, Angelova-Fischer I, Hoetzenecker W (2020). Hypersensitivity reactions to non-steroidal anti-inflammatory drugs: results of an Austrian cohort study. Allergo J Int.

[5] Greenberger PA (2019). Drug allergy. Allergy Asthma Proc.

[6] Doña I, Caubet JC, Brockow K, Doyle M, Moreno E, Terreehorst I (2018). An EAACI task force report: recognising the potential of the primary care physician in the diagnosis and management of drug hypersensitivity. Clin Transl Allergy.

[7] Wu JH-C, Langford BJ, Schwartz KL, Zvonar R, Raybardhan S, Leung V (2018). Potential negative effects of antimicrobial allergy labelling on patient care: a systematic review. Can J Hosp Pharm.

[8] Rubin R (2018). Overdiagnosis of penicillin allergy leads to costly, inappropriate treatment. JAMA.

[9] Blumenthal KG, Ryan EE, Li Y, Lee H, Kuhlen JL, Shenoy ES (2017). The impact of a reported penicillin allergy on surgical site infection risk. Clin Infect Dis.

[10] Chua KYL, Vogrin S, Bury S, Douglas A, Holmes NE, Tan N (2020). The penicillin allergy delabeling program: a multicenter whole-of-hospital health services intervention and comparative effectiveness study. Clin Infect Dis.

[11] Turner NA, Wrenn R, Sarubbi C, Kleris R, Lugar PL, Radojicic C (2021). Evaluation of a pharmacist-led penicillin allergy assessment program and allergy delabeling in a tertiary care hospital. JAMA Netw Open..

[12] Blumenthal KG, Oreskovic NM, Fu X, Shebl FM, Mancini CM, Maniates JM (2020). High-cost, high-need patients: the impact of reported penicillin allergy. Am J Manag Care.

[13] Wiboonsirikul K (2015). Development of repeated drug allergy prevention system in health promotion hospital network of Bang Pahan district, Phra Nakhon Si Ayutthaya province by evaluating the accuracy of patient drug allergic history. JPMAT.

[14] Cooper L, Harbour J, Sneddon J, Seaton RA (2021). Safety and efficacy of de-labelling penicillin allergy in adults using direct oral challenge: a systematic review. JAC Antimicrob Resist..

[15] Li L, Bensko J, Buchheit K, Saff RR, Laidlaw TM (2022). Safety, outcomes, and recommendations for two-step outpatient nonsteroidal anti-inflammatory drug challenges. J Allergy Clin Immunol Pract.

[16] Blumenthal KG, Peter JG, Trubiano JA, Phillips EJ (2019). Antibiotic allergy. Lancet.

[17] Staicu ML, Vyles D, Shenoy ES, Stone CA, Banks T, Alvarez KS (2020). Penicillin allergy delabeling: a multidisciplinary opportunity. J Allergy Clin Immunol Pract.

[18] Naranjo CA, Busto U, Sellers EM, Sandor P, Ruiz I, Roberts EA (1981). A method for estimating the probability of adverse drug reactions. Clin Pharmacol Ther.

[19] Brockow K, Przybilla B, Aberer W, Bircher AJ, Brehler R, Dickel H (2015). Guideline for the diagnosis of drug hypersensitivity reactions: S2K-Guideline of the German Society for Allergology and Clinical Immunology (DGAKI) and the German Dermatological Society (DDG) in collaboration with the Association of German Allergologists (AeDA), the German Society for Pediatric Allergology and Environmental Medicine (GPA), the German Contact Dermatitis Research Group (DKG), the Swiss Society for Allergy and Immunology (SGAI), the Austrian Society for Allergology and Immunology (ÖGAI), the German Academy of Allergology and Environmental Medicine (DAAU), the German Center for Documentation of Severe Skin Reactions and the German Federal Institute for Drugs and Medical Products (BfArM). Allergo J Int.

[20] Garvey LH, Ebo DG, Krøigaard M, Savic S, Clarke R, Cooke P (2019). The use of drug provocation testing in the investigation of suspected immediate perioperative allergic reactions: current status. Br J Anaesth.

[21] Wolfson AR, Banerji A (2021). Skin testing and drug challenge in the evaluation of drug hypersensitivity reactions. Allergy Asthma Proc.

[22] Barbaud A, Romano A (2022). Skin testing approaches for immediate and delayed hypersensitivity reactions. Immunol Allergy Clin N Am.

[23] Çelebioğlu E, Öztürk Aktaş Ö, Karakaya G, Kalyoncu AF (2021). A practical method of drug provocation testing to prove tolerance to alternative drugs in drug hypersensitivity. Turk J Med Sci.

[24] Soyer O, Sahiner UM, Sekerel BE (2017). Pro and contra: provocation tests in drug hypersensitivity. Int J Mol Sci.

[25] Abrams EM, Khan DA (2018). Diagnosing and managing drug allergy. CMAJ.

[26] Jarernsiripornkul N, Chaipichit N, Chumworathayi P, Krska J (2015). Management for improving patients' knowledge and understanding about drug allergy. Pharm Pract (Granada).

